# Duodenal-Jejunal Bypass Surgery Reverses Diabetic Phenotype and Reduces Obesity in *db/db* Mice

**DOI:** 10.2174/2213988501711010041

**Published:** 2017-10-31

**Authors:** Yongjun Liang, Yueqian Wang, Zhengdong Qiao, Ting Cao, Ying Feng, Lin Zhang, Peng Zhang

**Affiliations:** 1 Center for Medical Research and Innovation, Shanghai Pudong Hospital, Fudan University Pudong Medical Center, 2800 Gongwei Road, Pudong, , P.R. China; 2 Laboratory of Molecular Neuropharmacology, School of Pharmacy, East China University of Science and Technology, 130 Meilong Road, , P.R. China

**Keywords:** Type 2 Diabetes Mellitus (T2DM), Duodenal-Jejunal Bypass (DJB), *db/db* mice, Fasting Blood Glucose (FBG), Fasting Plasma Insulin

## Abstract

Type 2 diabetes mellitus (T2DM), a complex metabolic disorder typically accompanying weight gain, is associated with progressive β-cell failure and insulin resistance. Bariatric surgery ameliorates glucose tolerance and provides a near-perfect treatment. Duodenal-jejunal bypass (DJB) is an experimental procedure and has been studied in several rat models, but its influence in *db/db* mice, a transgenic model of T2DM, remains unclear. To investigate the effectiveness of DJB in *db/db* mice, we performed the surgery and evaluated metabolism improvement. Results showed that mice in DJB group weighed remarkably less than sham group two weeks after surgery. Compared to the preoperative level, postoperative fasting blood glucose (FBG) was dramatically reduced. Statistical analysis revealed that changes in body weight and FBG were significantly correlated. Besides, DJB surgery altered plasma insulin level with approximate 40% reduction. Thus, for the first time we proved that DJB can achieve rapid therapeutic effect in transgenic *db/db* mice with severe T2DM as well as obesity. In addition, decreased insulin level reflected better insulin sensitivity induced by DJB. In conclusion, our study demonstrates that DJB surgery may be a potentially effective way to treat obesity-associated T2DM.

## 1 INTRODUCTION

Type 2 Diabetes Mellitus (T2DM) and obesity, two types of metabolic homeostasis disorders, have become global epidemics. Currently, more than 400 million people suffer from diabetes globally and this number will reach 600 million in 2035 according to the International Diabetes Federation’s Diabetes Atlas sixth edition [1]. As a glucose homeostasis impaired disease, interactions between obesity and inadequate pancreatic β-cell response to the progressive insulin resistance result in T2DM. Obesity, another epidemic induced by disturbed energy-balance regulation, has influenced about 2 billion people worldwide [2]. Typically, obese patients having prediabetes are characterized with insulin resistance and at the same time 17% of these people will develop T2DM within 4 years. Thus, obesity is a high-risk factor for onset of T2DM. Although better lifestyle establishment and pharmaceutical interventions should be first-line and second-line treatment respectively for curing T2DM and obesity, success is difficult to achieve. Bariatric surgery, an emerging treatment, provides long-term remission of T2DM and sustained weight loss [3-5].

Roux-en-Y gastric bypass (RYGB), the most frequently used bariatric surgery, can achieve notably amelioration of T2DM in more than 80% of patients [6]. Especially, for the obesity-associated T2DM individuals, RYGB is the most powerful and effective solution both for diabetes itself and comorbid disease. Nonetheless, because of the complex rearrangement of gastrointestinal anatomy including stomach volume reduction and proximal small intestine bypass, RYGB can generate a certain amount of postoperative complications.

Duodenal-Jejunal Bypass (DJB), a stomach-sparing procedure of RYGB focused on intestinal function, was developed by Rubino and Marescaux to illustrate the mechanisms involved in glycemic control after surgery [7]. In a representative DJB surgery, the intact stomach is preserved and the entire duodenum and the proximal jejunum are bypassed simultaneously Fig. (**1**). Reconstructed gastrointestinal tract could be divided into three parts: Roux limb (providing expedited delivery of nutrients to the ileum), biliopancreatic limb (channel for bile and pancreatic juice passing), and the common channel (where food and bile/pancreatic juice mixed). Diabetic patients undergoing DJB will obtain improved glycemic control and other metabolic benefits [8-12]. In addition, DJB surgery in rats has revealed that glucose metabolism was enhanced independently of food intake or body weight in Goto-Kakizaki (GK) rats [7, 13], Zucker diabetic fatty (ZDF) rats [14] and streptozotocin-induced diabetic rats [15]. However, there was no study of DJB surgery reported in mice so far.

To investigate the therapeutic effects of DJB surgery in mice, *db/db* mice were employed in the present study. As a transgenic (leptin receptor deficient) model of T2DM, *db/db* mice has been widely used in diabetes research [16-18]. Considering the preservation of stomach, DJB surgery can massively reduce surgical morbidity and mortality so that we can concentrate on metabolic changes induced by intestinal bypass. In our work, by comparing preoperative and postoperative fasting blood glucose (FBG), body weight and fasting plasma insulin level, metabolic outcomes were assessed. The results may provide new insight in anti-diabetic capacity of DJB surgery.

## 2 MATERIALS AND METHODS

### 2.1 Animals

Male *db/db* mice aged 6 weeks were purchased from the Shanghai Research Center for Model Organisms (Shanghai, China). The mice were housed in a specific pathogen-free room at the research animal facility of Center for Medical Research and Innovation in Shanghai Pudong Hospital (Fudan Affiliated Pudong Medical Center). They were exposed to a strict 12-h light/dark cycle, constant ambient temperature (24±2°C) and humidity. Animals were allowed to acclimate for one week before starting the experiment and had free access to standard rodent food and sterile water. The study was approved by our Institutional Animal Experiment Committee in accordance with the guidelines.

### 2.2 Surgical Procedures

The mice were grouped randomly into DJB and sham groups. After overnight fasting, they were anesthetized with intraperitoneal injection of 3% pentobarbital sodium solution (0.2ml/100g). Abdominal skin was sterilized with iodophor disinfectant.

For mice in the DJB group, a midline abdominal incision through the skin and muscles was made 1.5 cm below the xiphoid to expose the pylorus and duodenum. The initial part of duodenum was tied off using double 5-0 silk threads. Nearby the ligation, the duodenum was transected 2mm distal to the pylorus. Next, the jejunum was divided 4~5cm below the ligament of Treiz and the distal end anastomosed to the pylorus in an end-to-end fashion with a single layer of interrupted stitches using 9-0 silk thread. Then, at about 4 cm distal to the site of gastrojejunal anastomosis, the side wall of the intestine was opened using a Vannas scissors, the biliopancreatic limb was anastomosed to the opening by interrupted stitches using 9-0 silk thread. After these procedures were completed, the abdominal was flushed with saline. The abdominal incision was closed with 5-0 silk thread followed by an intraperitoneal injection of 10% cefoxitin solution [7, 19].

For the mice in the sham group, celiotomy was performed to expose the pylorus and duodenum. The abdominal was irrigated with saline and the wound was closed with 5-0 silk thread. 10% cefoxitin solution was injected *via* intraperitoneal [20].

Mice were fasted on the day of surgery and fed with 5% glucose on the first postoperative day, a small amount of foods on the second postoperative day, and normal diet was fed afterwards.

### 2.3 Weight and Fasting Blood Glucose

After fasted for 6 hours, the mice were weighed by an electronic balance (Practum 2102-1S, Sartorius, Germany) and their fasting blood glucose were measured by a portable glucometer (ACCU-CHEK performa, Roche Diagnostics, USA) 3 days before surgery, one and two weeks after surgery.

### 2.4 Levels of Plasma Insulin

After overnight fasting, blood was collected from the tail vein of conscious mice into 1.5ml tubes before and 2 weeks after surgery. Blood samples were centrifuged at 3000rpm for 10 min at 4°C and serum was immediately separated and stored at -80°C until analysis. Enzyme-linked immunosorbent assay kits (Mercodia mouse insulin ELISA, Uppsala, Sweden) were used for measurement of insulin.

### 2.5 Statistical Analysis

Data were analyzed using the Statistical Package for Social Sciences software (version 22.0; SPSS Inc., Chicago, IL, USA) and expressed as mean ± SEM. Difference between DJB group and sham group were evaluated using Student’ *t*-test. Correlation analysis was processed between FBG and weight. *P* values < 0.05 were considered statistically significant.

## 3 RESULTS

### 3.1 Surgical Outcome

On the basis of pre-experiments, the anatomical structures involved in the DJB surgery were mastered proficiently and all operations were performed successfully. In the DJB group, the mean operative duration time was 67min (67±8.4min) and 5 mice survived with a success rate of 62.5% (5/8). Autopsy confirmed that one mouse died postoperatively because of anastomotic leakage and another two mice died due to intestinal obstruction. In the sham group, only one mouse died (5/6) of exhaustion and no complications were observed in the other mice through the whole process of the study.

### 3.2 Weight Loss After DJB Surgery

**Fig. (2):**
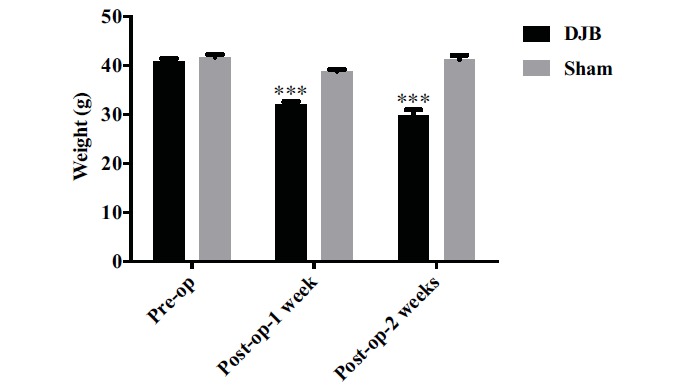
DJB surgery caused weight loss in *db/db* mice. Compared with the sham group, *db/db* mice in the DJB group had a significantly decline in body weight at two weeks postoperatively (***P<0.001).

Preoperative weight of DJB group and sham group were 40.9±0.6 and 41.7±0.6 g respectively and there was no statistical difference between the groups (*P*>0.05). Compared with sham mice, whose weights decreased slightly, DJB mice had approximate 22% loss in weight Fig. (**2**), ***P<0.001). Two weeks after surgery, the weight loss in *db/db* mice undergoing DJB surgery became even more significant Fig. (**2**), ***P<0.001). In addition, the body weight of mice in sham group regained rapidly and restored to their preoperative values at 2 weeks postoperatively (Fig. **2**).

### 3.3 Effect of DJB Surgery on Glycemic Control

Before surgery, no significant difference in FBG was noted between the two groups Fig. (**3**), *P*>0.05). Meanwhile, the basal line of FBG was too high to carry out the Oral Glucose Tolerance Test (OGTT). At the first week after the surgery, the mice in the DJB group demonstrated a rapid decline in FBG Fig. (**3**) and reached an almost normal level. Compared to the sham group, the FBG of DJB mice was dramatically decreased at 2 weeks postoperatively Fig. (**3**), ***P<0.001). Whether preoperative or postoperative, the FBG of sham group maintained high glucose levels stably.

### 3.4 Correlation Between Weight and FBG

Spearman rank correlation coefficient was calculated according to changes conducted by surgery in weight and FBG of mice in DJB group. The analysis indicated that the weight loss and FBG decline were significantly correlated with the correlation coefficient 0.721 Fig. (**4**), **P<0.05).

### 3.5 Insulin Responses to DJB Surgery

Due to the high glucose stimulation and low insulin sensitivity, preoperative plasma insulin concentration of DJB and sham group was approximate 1.4μg/L. DJB surgery caused remarkable reduction of plasma insulin levels in *db/db* mice in two weeks while mice in sham group remain the same status Fig. (**5**), ***P<0.001).

## 4 DISCUSSION

The present study investigated how DJB procedure affected metabolic homeostasis and obesity in *db/db* mice, a leptin receptor deficiency T2DM animal model. We confirmed that as a metabolic modification procedure by bypassing duodenum and proximal jejunum, DJB surgery is also beneficial toward resolving hyperglycemia and lowering body weight in this profound obese and T2DM animal model. This study implies that *db/db* mice can be a research model for bariatric surgery mechanistic studies.

Obesity associated-T2DM has been serious threat to human health and resulted in high social and economic costs. About 90% of T2DM is attributable to excess weight or obesity. Conversely, obese patients often accompany with prediabetes. Normally, lifestyle adjustment should be the first solution for the overweight or mildly obese patients. However, for severely obese patients with T2DM, pharmaceutical intervention is a must theoretically. Unfortunately, long-term glycemic control is difficult to achieve with any single oral hypoglycemic agent or any combination of agents [21]. Bariatric surgery brings new gospel and provides more efficient treatment in the prevention of T2DM in obese individuals. Although numerous studies have illustrated the mechanism with which the bariatric surgery functioned, there are lots of questions to be answered. This promoted us to carry out the work and seek more capacity of DJB surgery.

Based on the complexity of RYGB surgery comprising gastric and intestinal reconstruction and dissatisfactory postoperative complications, SG and DJB surgeries were invented alternatively focusing on the role of stomach and intestine. As a stomach-preserving model of RYGB, DJB has been proved to provide significant and durable glycemic control independently of food intake, body weight or nutrient absorption [7, 22]. According to the characteristics of DJB surgery, it has been recommended as an effective metabolic surgery for T2DM patients with lower body mass index (BMI) [23]. Clinically, patients with T2DM and a low BMI treated with DJB can achieve improved glycemic control [24-28]. In addition, DJB surgery in both obese and non-obese T2DM rat models such as GK [29-31], ZDF [14, 22] and high-fat diet combined streptozocin-induced diabetic rats [32-34] have been demonstrated remarkable improvements in glycemia. However, the therapeutic effect of DJB surgery in mice is still elusive until now and there was no study in this field according to our knowledge. In congruence with previous studies mentioned above, the *db/db* mice showed an amazing reduction in FBG at the first week postoperatively in our work. More interestingly, DJB surgery also caused notable weight loss correlated with FBG in the early stage after operation which was inconsistent with the results in rats. Because the *db/db* mice were executed at 2 weeks after surgery for a further study, long-term weight change wasn’t followed up. Based on this phenomenon, we speculated that it was an incipient consequence conducted by surgery [20] or different species or genetic background have distinct responses to DJB surgery.

Currently, the underlying mechanisms involved in anti-diabetic effects of DJB surgery are still not fully understood. Glucagon-like peptide-1 (GLP-1) secreted by L cells in the terminal ileum, mostly accepted as a mediator of glucose dependent insulin secretion [35], was considered to be a powerful prohibitor in preventing liver fat accumulation [34]. Another prevailing hypothesis suggests that the exclusion of duodenum and jejunum from nutrient transit might prevent the putative signals leading to insulin resistance [36-38]. Leptin, reduced by the DJB surgery in a diabetic rat model, is a proinflammatory adipokine which could lead to the improvement of insulin sensitivity [39]. In summary, ameliorative insulin sensitivity should be a critical contributor to the DJB surgery. As expected, plasma insulin level in DJB group reduced by about 40% postoperatively and this alteration may attribute to the improved glucose or insulin sensitivity. To understand the underlying mechanism, more work is needed.

In conclusion, although there is a tendency to abandon the DJB surgery clinically, it is still a powerful tool for T2DM or obesity research because of stomach-sparing. It can achieve a rapid and dramatic resolution of T2DM in *db/db* mice. Improved glycemic control was followed by striking weight loss at the early stage postoperatively. Finally, it is for the first time that we provide a novel demonstration that DJB surgery can reverse diabetic phenotype and improve obesity in *db/db* mice, which means *db/db* mice are costless and more effective model for metabolic surgery research.

## Figures and Tables

**Fig. (1):**
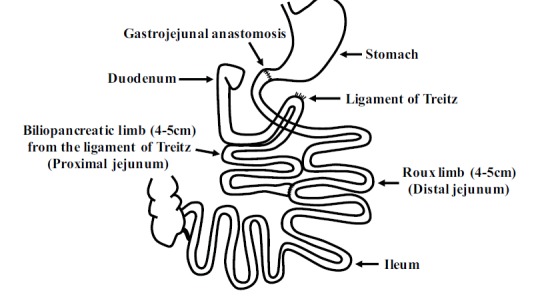
Schematic representation of duodenal-jejunal bypass surgery (DJB). Standard DJB surgery was adopted in our study. Duodenum and proximal jejunum (4-5cm distal to the ligament of Treiz) were bypassed. About 4 cm distal to the site of gastrojejunal anastomosis, the side wall of the intestinal was opened and anastomosed to the biliopancreatic limb.

**Fig. (3):**
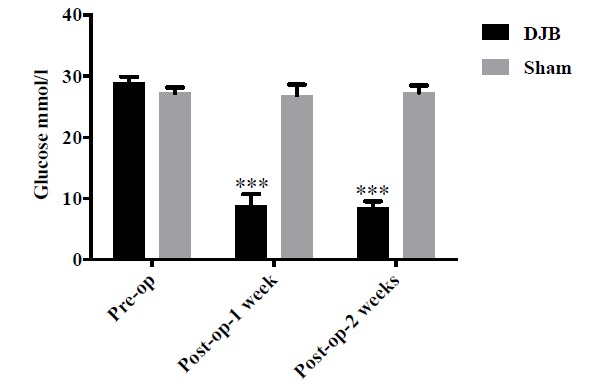
Glycemic control was improved by DJB surgery. Compared with the sham group, the FBG of mice undergoing DJB dramatically decreased at 1 week and 2 weeks after surgery (***P<0.001).

**Fig. (4):**
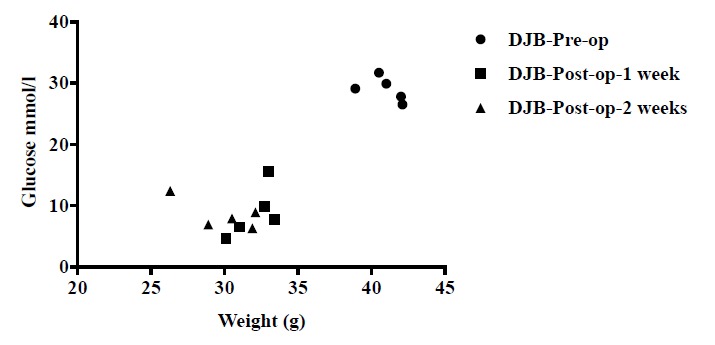
Correlation analysis of body weight and FBG. Surgery induced weight loss and FBG reduction was significantly correlated (**P<0.01).

**Fig. (5):**
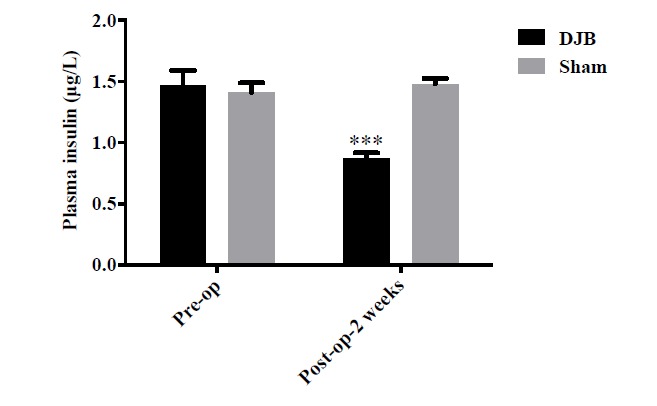
Effect of DJB surgery on plasma insulin level. The plasma insulin level of mice in the DJB group was 40% lower than the sham group (***P<0.001).
